# Provocation and prediction of visual peripersonal neglect-like symptoms in preoperative planning and during awake brain surgery

**DOI:** 10.1007/s00701-021-04822-2

**Published:** 2021-04-05

**Authors:** Viktória Tamás, Gabriella Sebestyén, Szilvia Anett Nagy, Péter Zsolt Horváth, Ákos Mérei, Francesco Tomaiuolo, Giovanni Raffa, Antonino Francesco Germanó, András Büki

**Affiliations:** 1 Department of Neurosurgery, Medical School, University of Pécs, Pécs, Hungary; 2 MTA-PTE Clinical Neuroscience MR Research Group, Pécs, Hungary; 3 János Szentágothai Research Centre, University of Pécs, Pécs, Hungary; 4 Pécs Diagnostic Center, Pécs, Hungary; 5 Department of Laboratory Medicine, Medical School, University of Pécs, Pécs, Hungary; 6 Department of Anesthesiology and Intensive Therapy, Medical School, University of Pécs, Pécs, Hungary; 7 Department of Clinical and Experimental Medicine, University of Messina, Messina, Italy; 8 Department of Biomedical, Dental, Morphological and Functional Imaging Sciences, University of Messina, Messina, Italy; 9 Neurotrauma Research Group, Szentágothai Research Centre, University of Pécs, Pécs, Hungary

**Keywords:** awake brain surgery, brain tumor, visual neglect, nTMS

## Abstract

Neglect is a severe neuropsychological/neurological deficit that usually develops due to lesions of the posterior inferior parietal area of the right hemisphere and is characterized by a lack of attention to the left side. Our case is a proven right-handed, 30-year-old female patient with a low-grade glioma, which was located in the temporo-opercular region and also in the superior temporal gyrus of the right hemisphere. Upon presurgical planning, the motor, language, and visuospatial functions were mapped. In order to achieve this, the protocol for routine magnetic resonance imaging and navigated transcranial magnetic stimulation has been expanded, accordingly.

**Supplementary Information:**

The online version contains supplementary material available at 10.1007/s00701-021-04822-2.

## Introduction

Recent clinical studies suggest the important role of supramaximal safe tumor resection in increasing life expectancy of patients with low-grade gliomas. During extended resective surgery, it is of utmost importance to spare functionally eloquent areas. This goal is best achieved by awake surgery, aided by intraoperative neuromonitoring and cognitive assessment. Neglect is a severe neuropsychological/neurological deficit characterized by the lack of attention for the left side of the space. It develops as a result of a lesion at the posterior section of the inferior-parietal region mostly of the right hemisphere [1, 6, 8].

## Case report

### Subject

A 30-year-old right-handed female has been diagnosed with a suspected low-grade glioma of the temporo-opercular region and superior temporal gyrus of the right hemisphere, affecting both cortical and subcortical areas. The patient has provided an informed consent acknowledging purported defect in the visual field.

### Mapping of the eloquent areas

Preoperative imaging workup included structural magnetic resonance imaging (MRI), diffusion tensor imaging (DTI) to evaluate white matter tracts and functional MRI (fMRI) to investigate neural correlates of the sensory language area.

Cortical mapping was performed with nTMS using Navigated Brain Stimulation system combined with the NEXSPEECH® module to locate the motor cortex, areas of language dominance, and those regions susceptible to neglect.

Although the patient was right-handed, language testing was necessary because the tumor was located in a Wernicke-homologue area, which (in a minor region) showed activation during comprehension of speech targeted fMRI testing. Mapping was performed on both hemispheres according to the previously published protocol [5] using trains consisting of 5 Hz/5 pulses, while the patient was performing an object naming test.

The examination of visuospatial capabilities was especially important because the tumor affected the neglect circle—although a less sensitive and less extensive region of it, while, in general, it was close to those structures which were widely associated with severe neglect syndrome [7].

During testing for neglect, 44 stimulation target points on the parietal region of the right hemisphere were defined, where a total of 140 stimuli were delivered while the patient was asked to evaluate the length of 80 differently mid-transected line (‘equal’; ‘left’ = left is longer; ‘right’ = right is longer). The pictures have been presented randomly. To aid the inhibitory effect a train of 10 Hz/5 pulses was used, with 100% of resting motor threshold.

### Visual and verbal stimuli

Pre- and intraoperative mapping of neglect-eloquent areas were performed by a line bisection judgment task, referred to as the landmark task according to descriptions [2–4].

**Fig. 1:**
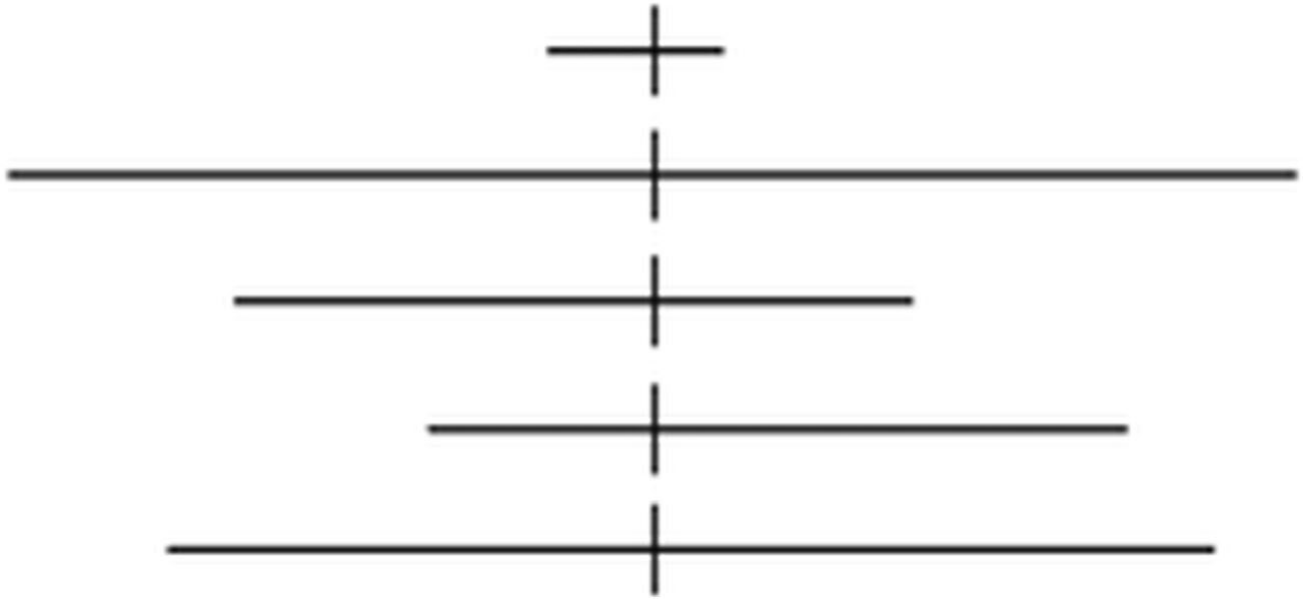
Line bisection judgment task, referred to as the landmark task to pre- and intraoperative mapping

Our own image library was built by the adaptation of NBS system. Horizontal black lines were split with a vertical line on a white background (Fig. 1).

Wrong judgment of length or the lack of answer were considered errors (neglect and non-neglect specific). Neglect-specific errors were categorized as follows: (1) longer left lines were named as equal; (2) longer left lines were named as longer right lines; (3) equal length lines were named as longer right. Non-neglect specific errors were categorized similarly: (1) longer right lines were named as equal; (2) longer right lines were named as longer left lines; (3) equal length lines were named as longer left.

### Neuropsychological evaluation

Mapping of neurocognitive and other psychological functions was performed 2 days prior to, as well as 4 and 5 days after surgery.

A variety of neuropsychological tests were performed for evaluating global neurocognitive functioning. (See Supplementary mat. 1)*.*

To assess visuospatial attention abilities and to detect neglect symptoms, line bisection test (visuospatial attention) and Bells Test (visuospatial attention) were included in our battery. These tests have been performed multiple times following surgery to monitor peripersonal visual neglect symptoms more closely.

## Results

### Preoperative phase

**Fig.2:**
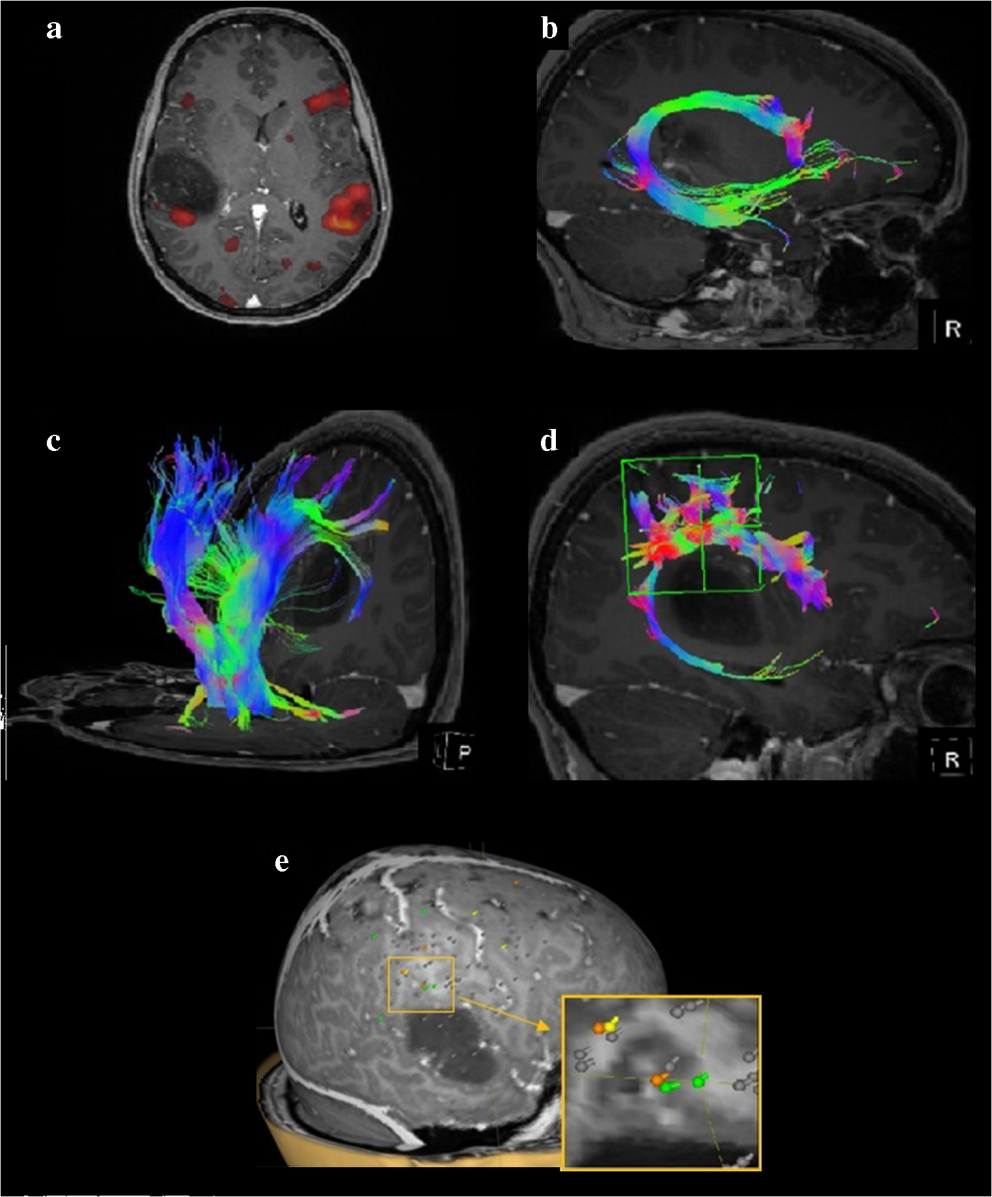
**a S**imultaneous left-sided dominant Wernicke activation and right-sided subdominant activity at the margin of the mass lesion. **b** Tractrogram of fiber network of FLS. **c** Tractrogram of fiber network representing the pyramidal tract. **d** Tractrogram of fiber network representing the tract system projected from the nTMS spots. **e** Errors in line bisection test during rTMS stimulation. Neglect specific errors are marked with yellow; non-neglect specific errors are marked with orange, delayed reaction times are marked with green

On fMRI, a right-sided subdominant activity appeared by the posterior-inferior margin of the lesion, besides the left-sided dominant Wernicke activation, which was not confirmed with nTMS (Fig. 2a).

Superior longitudinal fasciculus (FLS) and pyramidal tracts were intact by using DTI and fMRI—a finding confirmed by nTMS examination, too (Fig. 2b–d). A cluster of neglect-like and not neglect-specific spots (4 targets) were identified at the inferior parietal region (Table 1; Fig. 2e). These spots were used for tractographic analysis and for navigation during surgery. Furthermore, activation of other areas (6 instances, e.g., frontal lobe) was recognized, although more scattered than at previously mentioned areas.

**Table 1:** Parameters of neglect and non-neglect specified errors

Total line length (mm)	Left side (mm)	Right side (mm)	Error	Neglect (yes/no)
196	106	90	Delay	Yes
159	82	77	Equal	Yes
196	92	104	Equal	No
106	50	56	Equal	No

Significant neurological and neuropsychological disorder, which refers to contraindication in relationship with the examinations, could not be detected at the patient. Neglect-like symptoms have not been observed and motor functions seemed intact.

### Intraoperative phase

The asleep-awake-asleep operation was planned based on tractography and navigational MRI sequences. During the operation, frameless navigation-guided temporal craniotomy was performed; next functional areas were identified by bipolar fork probe using continuous cortical and subcortical stimulation. During intraoperative stimulation, we were not able to provoke speech error at the location (posterior portion of the superior temporal gyrus) according to nTMS results.

**Fig. 3:**
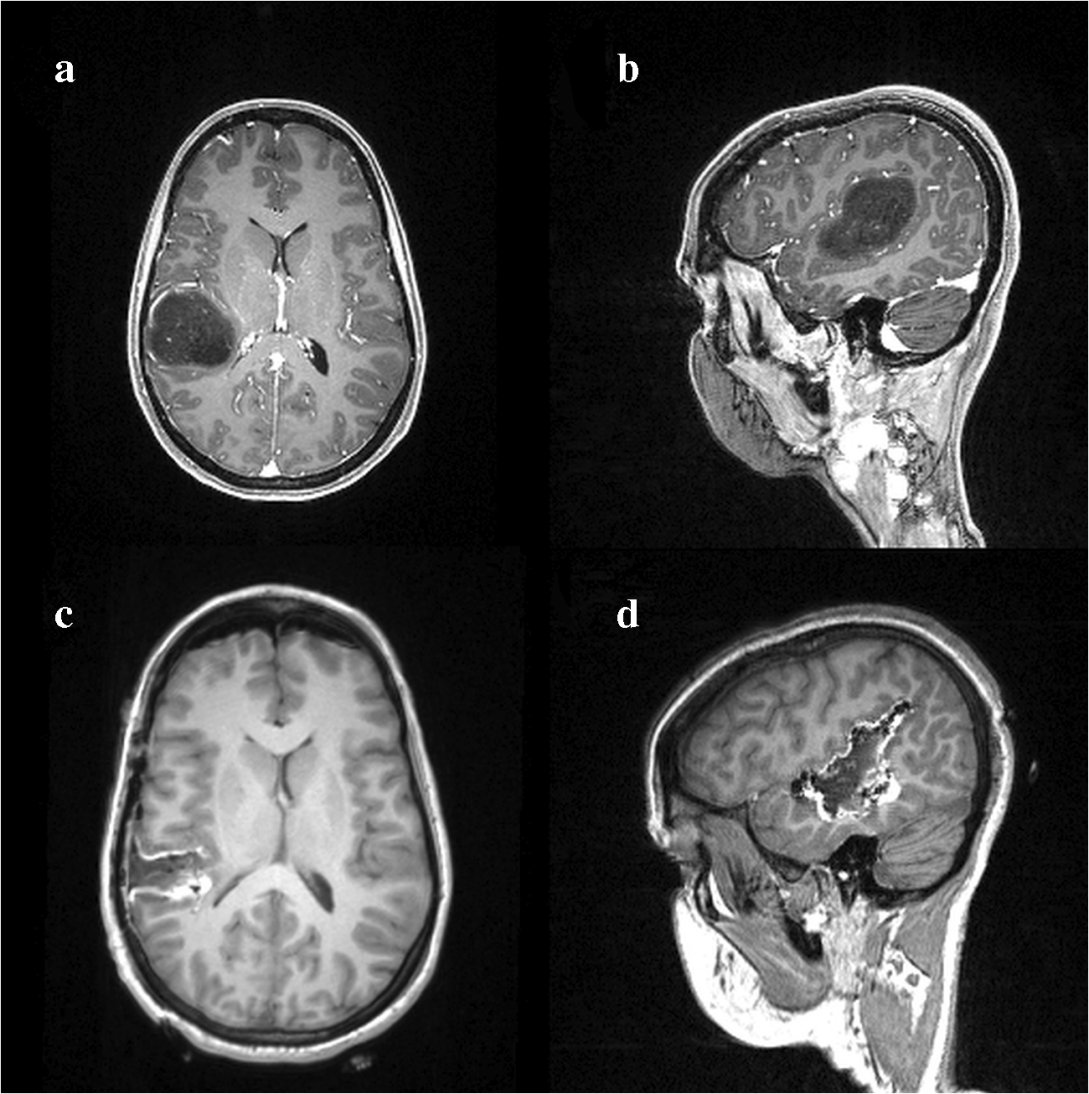
**a**, **b** T1-weighted contrast-enhanced MRI scan at preoperative phase demonstrate typical appearance of a low-grade glioma without any accumulation of the contrast medium. **c**, **d** T1-weighted contrast-free follow-up MRI scan 24 h after surgery revealed gross total removal of the tumor with thin hyperintense line indicative of minimal blood accumulation in the tumor bed

Initially, we barely found symptoms of neglect (1%) during intraoperative cortical mapping, while the rate of non-neglect specific errors was 22%. Contrarily, during the resection of the rear pole of the tumor, number of neglect specific errors increased significantly (+ 46%), while the number of non-neglect specific errors changed slightly (+ 3%). However, this location was further away from the area identified with nTMS as susceptible to neglect (inferior parietal lobule). Due to the transient manner of errors during the stimulation of this area, the resection was carried on. Using the acquired information, maximal safety was achieved during the resection. Postoperative MRI scan at 24 h confirmed total resection of the tumor (Fig. 3).

### Postoperative phase

#### MR imaging 24 h postoperatively

MRI showed postoperative changes with T1 hyperintensivity on the surface of the resection site as a sign of accumulated blood. FLAIR showed intensity of about 16 × 6 mm in size in the area constructing the lateral wall of the posterior horn the right lateral ventricle. Presence of some postoperative air in front of the frontal poles was described.

#### Neuropsychology

Twenty-four hours after the surgery a moderate ego- and allocentric visual neglect has been detected peripersonally, for the left side of the space, which was clearly observed in the behavior of patient (e.g., the bisection of horizontal lines were happened from right side to the left side) and also in the recorded tests (an obvious, average orientation of 2.5 cm for right side in the line-bisection test, and weaker performance with 40% in the Bells test due to the omission of the left elements). The patient lies on the right side of the bed, despite the fact that the surgical scar was also located on the right side of her head. Nevertheless, personal and/or extrapersonal neglect syndrome was not perceived by the clinical observation.

On the fourth day following the operation (more than 72 h later), visuospatial capabilities show a significant improvement (number of identified elements in the left visual filed was increased with 17% in the Bells test); however, vision problems are still indicated by the patient (“Something is weird”); therefore, a confrontational visual field exam was completed based on the Dean-Woodcock Neuropsychological Battery. Complete right and left side, as well as simultaneous (concomitant, extinction) testing of the upper, middle, and lower visual fields shows partial left homonymous hemianopia in the patient (total left: point = 0, “*w*” value = 415/*severe*; total simultaneous: point = 0, “*w*” value = 429/*severe*; total right: point = 24, “*w*” value = 488/*normal zone*).

It is of note that on the basis of surgical planning based on preoperative assessment of optic radiation as well as postop MR images such partial visual field defect could well have been anticipated upon gross total tumor resection.

On the fifth day, the homonymous hemianopia was still detected, while the symptoms (and behavior) referred to the neglect completely disappeared. The Bells test has already performed correctly, with similar search strategy as before the surgery, and no significant difference was observed between the length of horizontal lines that have been halved in the line-bisection task.

**Fig. 4:**
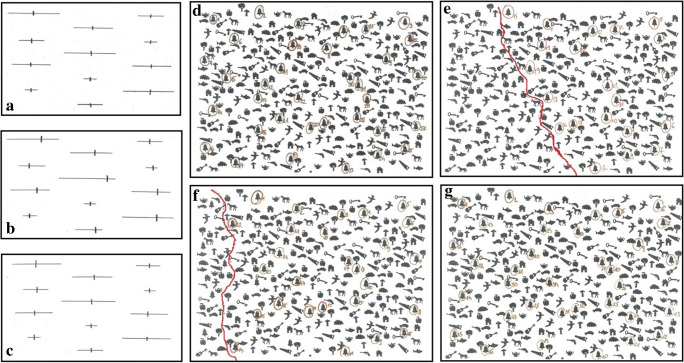
The alteration in visuospatial attention functions (the appearance of neglect-like symptoms). **a** Line bisection test—preoperative phase. **b**, **c** Line bisection test—postoperative phase. **d** Bells test—preoperative phase. **e**–**g** Bells test—postoperative phase

Regarding the other neuropsychological functions, the pre- and postoperative results were summarized in Table 2. The alteration in visuospatial attention functions was illustrated in Fig. 4.

**Table 2:** Results of psychological tests

	Preoperative assessment	Postoperative assessment	Cut-off scores
RAVLT1	7	6	6.7
RAVLT2	9	9	9.9
RAVLT3	9	10	11.4
RAVLT4	11	12	12.2
RAVLT5	12	11	12.7
RAVLTB	4	3	6.5
RAVLT6—immediate recall	11	8	11.2
RAVLT7—delayed recall	8	10	11.1
RAVLT—recognition (correct answers)	15 (+ 9 false intruses)	13 (+ 0 false intruses)	14.2
Five-point test (correct patterns)	13	16	> 10
Five point test—number of perseverations	0	0	< 2
Verbal fluency task—phonological part (FAS)—SUM	38	40	≥ 30
Verbal fluency task—semantic part (animal, vehicle, vegetable)—SUM	52	51	≥ 50
Pieron (T%)	90,9	87,3	98,9
Pieron (N)	287	269	345
Digit span—forward	5	6	> 5
Digit span—backward	3	3	> 4
TMT A (s)	46	48	24.40
TMT B (s)	50	77	50.68
FAB	16	18	12
Clock drawing test	10	10	9
Rey-Osterrieth complex figure test—copying part	35	27	≤ 32
Rey-Osterrieth complex figure test—immediate recall	17,5	17,5	≤ 22
DWNB—picture naming /*W* value	549	561	540
DWNB—finger tapping/dominant/*W* value	512 (normal)	504 (normal)	508
DWNB—finger tapping/non-dominant/*W* value	515 (normal)	514 (normal)	508
DWNB—strength of grip/dominant/*W* value	525 (normal)	523 (normal)	530
DWNB—strength of grip/non-dominant/*W* value	524 (normal)	523 (normal)	528
Beck anxiety inventory	5	23	> 21
Beck depression inventory	3	7	> 4
SF-12	50	27	< 48
MD Anderson symptom inventory	10	63	> 14

## Conclusion

In this work, we have identified a complex pre-, intra-, and postoperative protocol for the assessment of neglect in conjunction with awake surgery upon gross total removal of a low-grade glioma. Navigated—MRI-based—transcortical magnetic stimulation has been included as integrated part of the preoperative workup and planning.

According to the pre- and intraoperative assessments and the results presented in this case, the applied paradigm was useful for identifying eloquent areas of visuospatial attention and had predictive value for the development of postoperative neglect-like symptoms. In addition to neuromonitoring, this was further improved by the surgical strategy followed in the intraoperative. Our case report supports the literature data that lesions of the superior temporal gyrus play less role in the appearance of permanent neglect-like symptoms, but rather can result in transient changes. The paradigms established here should aid supramaximal safe resection of tumors aiding prolonged survival and better quality of life of our patients.

## Supplementary Information

ESM 1 (PDF 306 kb)
