# Supplementary material for: Provocation and prediction of visual peripersonal neglect-like symptoms in preoperative planning and during awake brain surgery
Source: Acta Neurochir (Wien). 2021 Apr 5;163(7):1941–7. doi: 10.1007/s00701-021-04822-2 (PMC8195910; doi:10.1007/s00701-021-04822-2)
Supplement: Supplementary file 1 — (PDF 306 kb) [file 701_2021_4822_MOESM1_ESM.pdf]

Provocation and prediction of visual peripersonal neglect-like symptoms in preoperative planning and during awake surgery

Viktória Tamás[1]
Gabriella Sebestyén [1]
Szilvia Anett Nagy [2,3,4,5]
Péter Zsolt Horváth [1]
Ákos Mérei [6]
Francesco Tomaiuolo [7]
Giovanni Raffa [8]
Antonino Francesco Germanó [8]
András Büki [1,9]

[1]Department of Neurosurgery, Medical School, University of Pécs, Hungary,
[2]MTA-PTE Clinical Neuroscience MR Research Group, Pécs, Hungary, Hungary
[3]János Szentágothai Research Centre, University of Pécs, Hungary
[4]Pécs Diagnostic Center, Hungary
[5]Department of Laboratory Medicine,Medical School, University of Pécs, Hungary
[6]Department of Anesthesiology and Intensive Therapy, Medical School, University of Pécs, Hungary
[7]Department of Clinical and Experimental Medicine, University of Messina, Italy
[8]Department of Biomedical, Dental, Morphological and Functional Imaging Sciences, University of Messina, Italy
[9]Neurotrauma Research Group, Szentágothai Research Centre, University of Pécs, Hungary

Corresponding Author: Viktória Tamás, tamas.viktoria@pte.hu, +36305594559

Supplementary Material 1. Tests for neuropsychological/psychological evaluation

- Rey Auditory Verbal Learning Test (RAVLT) – verbal learning and episodic memory
- Phonemic and semantic Verbal Fluency Tests - executive functions and semantic retrieval\retrograde memory
- Rey Osterrieth Complex Figure Test (ROCF) visual attention, incidental memory, executive functions
- Part A and B of the Trail Making Test (TMT A and B) - psychomotor speed, visuomotor skills, mental flexibility, attention switching
- Frontal Assessment Battery (FAB) - executive functions, inhibitory control
- Digit Span Forward and Backward - short-term memory/attention and working memory)
- Pieron Test – attention, concentration
- Clock-Drawing Test - visuospatial abilities, executive functions
- Picture naming part of the Dean Woodcock Neuropsychological Battery (DWNB) – language, gnosis
- Finger Tapping and Grip Strength Assessment of Dean Woodcock Neuropsychological Battery (DWNB) – motor functions/skills
- Pattern fluency test (Five-Point Test) – executive functions, visual attention
- Shortened Beck Depression Inventory (13 items), Beck Anxiety Inventory, SF-12 Questionnaire and the MD Anderson Symptom Inventory - assessment of intrapsychic and health state
- Line Bisection Test – visuospatial attention, vision
- Bells test – visuospatial attention, vision
